# Preemptive Rituximab for Epstein–Barr Virus Reactivation After Hematopoietic Cell Transplantation: Necessary for All?

**DOI:** 10.1111/tid.70124

**Published:** 2025-11-03

**Authors:** Anna Beatriz Coelho de Souza, Anderson João Simione, Ana Cláudia Ferrari dos Santos, Iago Colturato, Fernanda Rodrigues Barbieri, Juliana Ribeiro do Prado Moreno, Lilian Perílio Zanetti, Leila Cibele Serra de Oliveira, Erika Rodrigues Pontes Delattre, Juliana Silva Santos, Mair Pedro de Souza, Vergílio A. R. Colturato, Clarisse M. Machado

**Affiliations:** ^1^ HCT Program Fundação Hospital Amaral Carvalho Jaú Brazil; ^2^ Virology Laboratory Institute of Tropical Medicine Faculty of Medicine University of São Paulo São Paulo Brazil

**Keywords:** EBV, end‐organ disease, HCT, immunosuppression, PTLD, rituximab

## Abstract

**Introduction:**

In allogeneic HCT recipients, risk factors for PTLD and EBV end‐organ diseases are well established. Therefore, weekly monitoring of EBV reactivation with quantitative PCR is indicated for patients at risk. Although based on uncontrolled studies and supported by moderate strength of evidence, preemptive rituximab has been recommended in cases of EBV reactivation, without a clearly defined EBV DNAemia threshold. Rituximab is known to be associated with prolonged B‐cell depletion and secondary hypogammaglobulinemia, resulting in an increased risk of infectious complications and poor vaccine responses for an extended period.

**Methods:**

In this retrospective single‐center study, we evaluated the safety and effectiveness of immunosuppression (IS) reduction as the first approach in EBV reactivation in 328 HCT recipients, limiting the introduction of rituximab to patients who did not respond to IS reduction or who developed EBV end‐organ disease or PTLD.

**Results:**

During follow‐up, 178 patients experienced EBV reactivation, with a cumulative incidence of 54.6%. Among these, four patients developed EBV encephalitis (2.2%), and no cases of PTLD were identified. Rituximab was administered to only 12 patients (6.7%). In multivariate analysis, EBV reactivation was significantly associated with chronic GVHD, which may be related to EBV reactivation itself, rapid IS withdrawal, or both. EBV reactivation did not adversely affect non‐relapse mortality or overall survival in this cohort.

**Conclusion:**

IS reduction as the first‐line approach to EBV reactivation was safe and effective in most patients with increasing EBV DNAemia. Consequently, rituximab was required in fewer than 10% of cases.

## Graphical abstract

A sudden increase in EBV DNAemia appears to be a better predictor of PTLD and EBV end‐organ disease than persistent and stable levels of EBV DNAemia. Most HCT recipients with EBV DNAemia did not require rituximab, and immunosuppression reduction was sufficient to control most episodes of EBV reactivation.

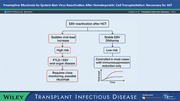

## 1 Introduction

Epstein–Barr virus (EBV)‐related post‐transplant lymphoproliferative disorder (PTLD) is a significant cause of morbidity and mortality in hematopoietic cell transplant (HCT) recipients. The risk of EBV‐PTLD is primarily associated with the degree of T‐cell depletion. Well‐recognized pre‐transplant risk factors include donor/recipient EBV serology mismatch, cord blood transplantation (CBT), HLA mismatch, second HCT, and the use of anti‐thymocyte globulin (ATG) or alemtuzumab. High‐risk criteria for PTLD include matched or mismatched unrelated donor (MUD/mMUD) transplantation, alternative donors such as CBT, and matched related donor (MRD) HCT with at least one additional risk factor [1].

Prospective monitoring of EBV DNAemia by quantitative PCR (qPCR) is recommended in high‐risk patients. Pooled data from published studies in HCT recipients suggest that preemptive administration of rituximab, a chimeric anti‐CD20 monoclonal antibody, leads to favorable outcomes in approximately 90% of patients and in 65% of those with established EBV‐PTLD [1]. Along with rituximab, immunosuppression (IS) reduction is also recommended, when feasible [2].

Because IS impairs T‐cell function, reducing IS can restore EBV‐specific cytotoxic T‐cell activity and, consequently, lower EBV DNAemia. Rituximab, by contrast, causes rapid depletion of CD20‐positive B cells in peripheral blood, with delayed recovery, leading to secondary hypogammaglobulinemia, impaired humoral response, increased susceptibility to infections, and poor vaccine responses for extended periods [3, 4]. In a large cohort study of adult patients treated with rituximab for various indications, 19% of those with normal baseline immunoglobulin levels developed mild to severe deficiency after 18 months [5]. During the COVID‐19 pandemic, several studies demonstrated that patients who had received rituximab were at increased risk of COVID‐19 complications, had reduced long‐term immunity, and showed impaired vaccine responses [6, 7]. Therefore, unnecessary use of rituximab should be avoided whenever possible.

In Brazil, rituximab availability is limited in public health services. Consequently, active surveillance of EBV viral load (VL) dynamics and IS reduction represent the main strategies for EBV control in high‐risk HCT recipients at our center.

We hypothesized that if this strategy were unsafe or ineffective, patients with EBV reactivation not treated with rituximab would experience lower overall survival (OS) and higher non‐relapse mortality (NRM) compared with those without EBV reactivation. In addition, the incidence of EBV‐related complications (PTLD and EBV end‐organ disease) would exceed that reported in the literature. To test this, we conducted a retrospective cohort study to evaluate whether reducing IS as the primary intervention for EBV reactivation could safely and effectively prevent PTLD and EBV‐related complications.

## 2 Methods

### 2.1 Study Design and Population

This was a retrospective, single‐center cohort study. Between January 2017 and December 2021, 328 consecutive allogeneic HCT recipients considered at high risk for PTLD were included. Fifty‐four patients (16.4%) underwent a second HCT. The study was conducted at the HCT Program of Hospital Amaral Carvalho, currently the largest public HCT center in Brazil.

### 2.2 Definitions

Risk factors for EBV‐PTLD were defined according to ECIL‐6 guidelines: T‐cell depletion (in vivo or ex vivo), donor/recipient EBV serology mismatch, CBT, HLA mismatch, splenectomy, second HCT, severe acute or chronic GVHD, high or rising EBV viral load, and treatment with mesenchymal stem cells. High‐risk criteria were defined as follows: Recipients of MRD with at least one risk factor, mismatched related donors (mMRD), MUD, mMUD, or recipients treated with ATG [1]. EBV reactivation was defined as the detection of EBV DNAemia at any level [8, 9]. EBV end‐organ disease and PTLD were classified according to the sixth European Conference on Infections in Leukemia (ECIL‐6) criteria [1].

### 2.3 EBV Policies

Donor and recipient EBV serology was performed irregularly and therefore not included in this analysis. The need for EBV monitoring by quantitative PCR (qPCR) was determined during the pre‐transplant evaluation, according to high‐risk criteria. EBV DNAemia was monitored weekly using qPCR (Master kit for Epstein–Barr virus quantification, Mobius Life Science, Pinhais, PR, Brazil). Whenever EBV viral load increased by ≥1 log within one week, a laboratory alert was issued to the clinical team, who promptly initiated IS reduction (when feasible) and performed an EBV workup (evaluation for EBV end‐organ disease or PTLD). IS reduction was performed at the physician's discretion, on a case‐by‐case basis, considering time since HCT, disease status, GVHD status and control, among other clinical factors. The EBV workup included a detailed physical examination and imaging studies (ultrasound, computed tomography, or magnetic resonance imaging). In the presence of neurological symptoms, cerebrospinal fluid (CSF) was analyzed. Rituximab (375 mg/m^2^, 1–2 weekly doses) was restricted to asymptomatic patients who failed IS reduction (preemptive rituximab) or to patients who developed EBV end‐organ disease or PTLD. In Brazil's public health system, rituximab is available only for patients with rheumatoid arthritis, non‐Hodgkin lymphoma, large B‐cell lymphoma, follicular lymphoma, and chronic lymphocytic leukemia.

### 2.4 Statistical Analysis

The incidence of EBV reactivation, NRM, and relapse was estimated using cumulative incidence methods (R software). Non‐parametric variables were compared using the Mann–Whitney test. Multivariate analysis was performed with Fine–Gray hazard regression for competing risks. OS was estimated using the Kaplan–Meier method and compared between patients with or without EBV reactivation using the log‐rank test (SPSS version 21).

### 2.5 Ethics

The study is part of a study of viral biomarkers in HCT recipients and the procedures followed the ethical standards of Helsinki declaration. The local Ethics Committee has approved the study, which was registered as CAAE number 49831221.0.0000.5434 at Brazil Platform, the national database of research records in humans.

## 3 Results

### 3.1 EBV Reactivation

EBV reactivation was detected in 178 patients (114 MUD and 64 haploidentical) at a median of 53 days after HCT (range, 3–439). Patient characteristics are summarized in Table 1. The cumulative incidence of EBV DNAemia was 54.6% (95% CI, 49–59.9) and was significantly higher after MUD compared with haploidentical HCT (73.1% vs. 37.2%; *p* < 0.001). In univariate analysis, MUD HCT, ATG exposure, and peripheral blood stem cells (PBSC) as the graft source were significantly associated with EBV reactivation. In multivariate analysis, only ATG use remained significantly associated (Table 2).

**TABLE 1:** Patients’ characteristics (N=328)

Variables		*N* (%)
**Median age at HCT (range)**		21 (1–66) years
**Age group (years)**	0‐17	130 (39.6)
	18 ‐ 40	129 (39.3)
	>40	69 (21)
**Gender**	Female	129 (39.3)
	Male	199 (60.7)
**Donor**	Haploidentical	172 (52.4)
	Unrelated	156 (47.6)
**Underlying diseases**	Acute leukaemia	214 (65.3)
	Lymphoma	16 (4.9)
	Non‐oncological	33 (10)
	Myelodysplastic syndrome	30 (9.1)
	Others	35 (10.6)
**Performance Status**	90‐100%	297 (90.5)
	<90%	31 (9.5)
**Donor Gender**	Female	85 (25.9)
	Male	243 (74.1)
**Source of stem cells** *	PBSC	85 (26)
	BM	242 (74)
**Conditioning**	NMA/RIC	132 (40.2)
	MA	196 (59.8)

*Data not available in one patient.

Abbreviations: BM, bone marrow; NMA, non‐myeloablative; PBSC, peripheral blood stem cell; RIC, reduced intensity conditioning; MA, myeloablative.

**TABLE 2:** Univariate and multivariate analysis of variables associated with EBV reactivation.

Variables	Univariate			Multivariate		
	HR	95%CI	*p*	HR	95%CI	*p*
Age at HCT	1.00	1.00‐1.01	0.360			
18 to 40 years	1.05	0.75‐1.46	0.790			
>40 years	1.22	0.82‐1.82	0.320			
Male sex	1.06	0.78‐1.44	0.700			
MUD HCT	3.68	2.69‐5.01	<0.001	0.31	0.04‐2.12	0.230
2^nd^ HCT	0.88	0.58‐1.34	0.550			
Performance status <90%	1.24	0.77‐2.00	0.370			
UD lymphomas	0.53	0.22‐1.30	0.160			
UD non‐oncological	1.45	0.91‐2.30	0.120			
UD others	0.96	0.65‐1.40	0.830			
Male donor	1.21	0.86‐1.71	0.270			
ATG in GVHD prophylaxis	3.28	2.40‐4.47	<0.001	9.81	1.47‐65.38	0.018
Stem cell source BM	0.57	0.42‐0.79	<0.001	0.95	0.67‐1.36	0.810
MA conditioning	0.89	0.66‐1.21	0.470			

Abbreviations: BM, bone marrow; GVHD, graft versus host disease; MUD, matched unrelated donor; MA, myeloablative UD, underlying disease.

### 3.2 Acute and Chronic GVHD

The median onset of acute GVHD was 33 days (range, 8–250), and chronic GVHD was 165 days (range, 28–1001) post‐HCT. The cumulative incidence of Grade II–IV acute GVHD was 27% (95% CI, 20.7–33.7) among patients with EBV reactivation and 18.7% (95% CI, 12.9–25.3) among those without reactivation (*p* = 0.072). Chronic GVHD was more frequent in patients with prior EBV reactivation (36.7% vs. 24.1%, *p* = 0.014). In multivariate analysis, factors associated with chronic GVHD included donor age > 40 years (HR 1.71; 95% CI, 1.11–2.63; *p* = 0.014), male sex (HR 1.79; 95% CI, 1.18–2.70; *p* = 0.005), prior acute GVHD (HR 1.52; 95% CI, 1.00–2.30; *p* = 0.045), second HCT (HR 1.65; 95% CI, 1.03–2.64; *p* = 0.037), and EBV reactivation (HR 1.70; 95% CI, 1.10–2.63; *p* = 0.016) (Table 3).

**TABLE 3:** Multivariate analysis of variables associated with chronic GVHD.

Variable	Multivariate		
	HR	95%CI	*p*
Donor age > 40 years	1.71	1.11‐2.63	0.014
Donor gender (male)	1.79	1.18‐2.70	0.005
Previous acute GVHD	1.52	1.00‐2.30	0.045
Second HCT	1.65	1.03‐2.64	0.037
EBV reactivation	1.70	1.10‐2.63	0.016

### 3.3 Rituximab

**FIGURE 1:**
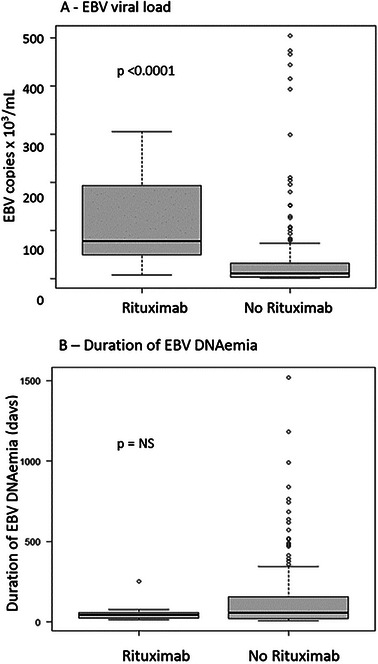
EBV viral load (A) and duration of EBV DNAemia (b) according to rituximab use.

According to institutional EBV management policies, rituximab was administered to 12 patients (6.7%). Indications included failure to respond to IS reduction (*n* = 7) and EBV encephalitis diagnosed during EBV workup (*n* = 4). One additional patient received a single dose on Day +98 due to suspected EBV encephalitis without prior EBV DNAemia. This diagnosis was later excluded after CSF retesting at a reference laboratory. The median EBV viral load in patients receiving rituximab was 78,232 copies/mL (range, 8335–305,295), significantly higher than in those not receiving rituximab (median, 11,450; range, 1000–504,555; *p* < 0.0001). The duration of EBV DNAemia was similar between patients treated with rituximab and those who were not (median 41 days, range 14–252 vs. median 56 days, range 6–1,520; Figure 1).

### 3.4 EBV Encephalitis

**FIGURE 2:**
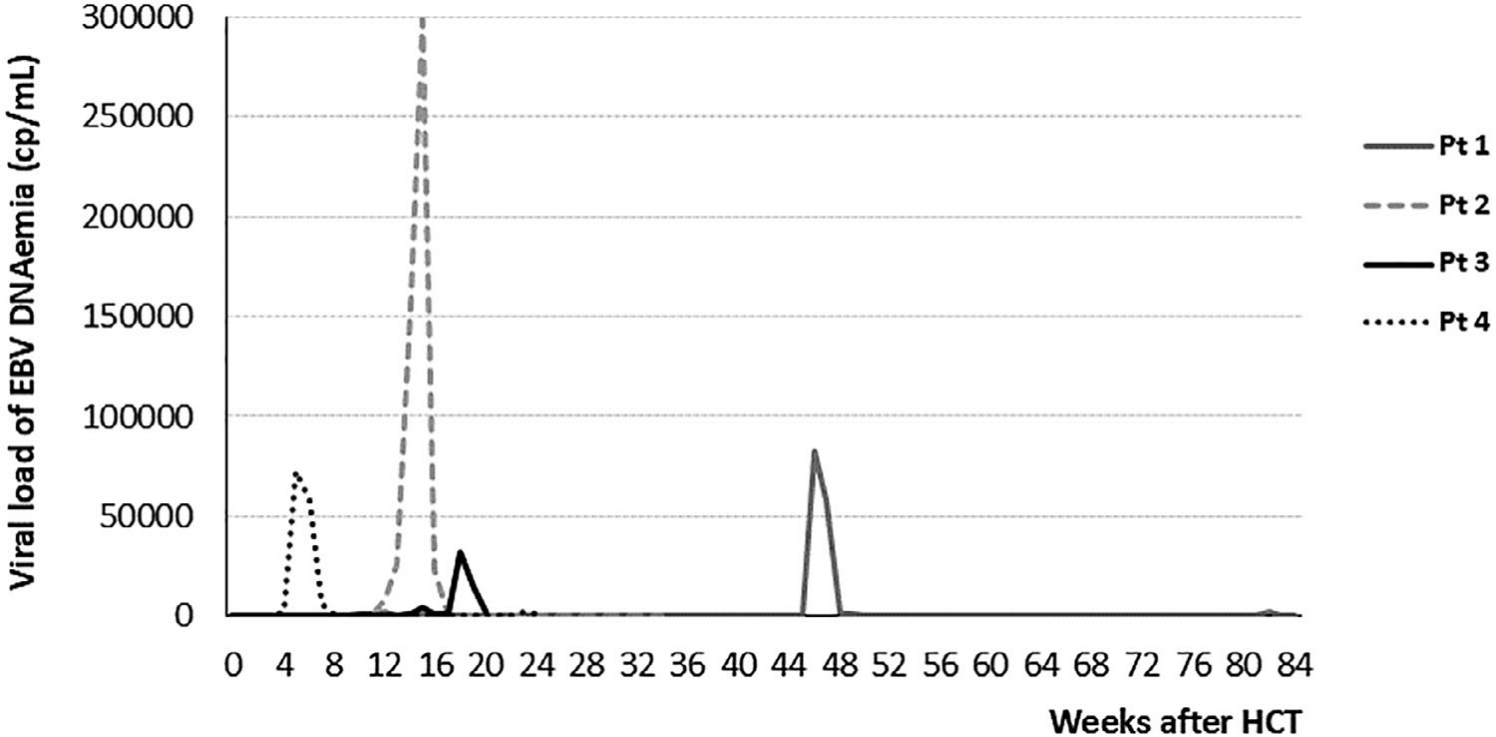
Dynamics of EBV DNAemia in encephalitis cases.

Among patients with EBV reactivation, EBV encephalitis was diagnosed in 4 cases (2.2%), based on neurological symptoms and EBV detection in CSF by PCR. In all cases, encephalitis was preceded by an abrupt rise in EBV DNAemia (Figure 2). Three of four patients (75%) responded to rituximab, showing immediate declines in EBV viral load. The median peak EBV DNAemia before encephalitis was 77,617 copies/mL (range 32,180–298,370). CSF EBV viral load was lower, with a median of 1707 copies/mL (range 1145–2,555). Peak viral load did not correlate with outcomes. The patient with the lowest DNAemia peak (32,180 copies/mL) died from EBV encephalitis, whereas two patients with the highest peaks (82,470 and 298,370 copies/mL) survived and remain alive and well more than four years later. Patient 2 who had the highest peak of EBV DNAemia had received a MUD HCT with myeloablative conditioning and ATG due to a myelodysplastic syndrome. She presented with a sudden neurological condition on Day +112, with fever, seizures, and mental confusion. EBV viral load in CSF was 2060 cp/mL. The fourth patient improved after rituximab with clearance of EBV DNA from CSF but died four months later from unrelated infectious complications.

### 3.5 PTLD

No cases of PTLD were identified. One patient developed a monoclonal proliferation of large granular lymphocytes (mostly CD8+) during a complicated course of CMV retinitis and recurrent CMV viremia. Concurrent EBV DNAemia at low levels (2655 copies/mL) was detected without evidence of EBV end‐organ disease or PTLD. The patient cleared EBV DNAemia spontaneously, did not receive rituximab, and remains alive four years later.

### 3.6 OS and NRM

EBV reactivation did not impact OS or NRM. OS was 71.5% in patients with EBV reactivation versus 59.7% in those without (*p* = 0.078). NRM was 15.2% versus 15.0%, respectively (*p* = 0.836).

## 4 Discussion

The cumulative incidence of EBV DNAemia in this study (54.6%) falls within the range reported in the literature. Rates of EBV reactivation vary widely across transplant centers, from 18.6% to 81.7%, depending on factors such as EBV seroprevalence, transplant type, patient risk profile, assay sensitivity, viral load thresholds, and the duration of monitoring [10].

In our cohort, only ATG exposure remained independently associated with EBV reactivation in multivariate analysis. A previous study from the Infectious Diseases Working Party of the European Group for Blood and Marrow Transplantation (EBMT) reported lower PTLD incidence after MRD and mismatched related donor (mMRD/haploidentical) transplants compared with MUD transplants [11].

Monitoring EBV DNAemia in high‐risk patients is undoubtedly essential. However, it is important to recognize that the positive predictive value of high viral load for EBV‐related complications remains low, even in high‐risk populations. Consequently, current recommendations for preemptive rituximab in the setting of EBV reactivation—without a defined DNAemia threshold—may result in long‐term impairment of immune reconstitution in patients not truly at risk [9]. Conversely, the negative predictive value of the absence of EBV reactivation is excellent, allowing safe follow‐up without intervention [9, 12].

We hypothesized that patients with rapidly rising EBV DNAemia would be at greater risk of EBV end‐organ disease or PTLD, and would therefore require IS reduction. Conversely, patients with low and stable EBV DNAemia could be monitored closely without intervention.

As expected, viral loads were higher in patients who failed IS reduction and subsequently required rituximab compared with those who responded to IS tapering and avoided rituximab (Figure 1A). However, the duration of DNAemia did not correlate with severity, being similar between patients who did and did not receive rituximab (Figure 1B). Taken together, these findings suggest that a sudden ≥1‐log increase in viral load within one week—rather than the persistence of DNAemia at stable levels—may be a better biomarker for predicting PTLD or EBV end‐organ disease risk, as proposed by other authors [10, 13]. We observed that all cases of EBV encephalitis were preceded by an abrupt increase in EBV DNAemia, exceeding 26,000 copies/mL (Figure 2). Unexpectedly, the absolute peak viral load did not predict outcomes. For example, the patient with the lowest peak (32,180 copies/mL) died, whereas the two patients with the highest peaks (82,470 and 298,370 copies/mL) survived long‐term. This suggests that additional host or transplant‐related factors influence prognosis.

The reported frequency of EBV encephalitis/myelitis after HCT ranges from 1.2% to over 12%. In a cohort of 39 allogeneic HCT recipients, Kinch et al. observed EBV reactivation in 16 patients (41%), two of whom (12.5%) developed neurological manifestations [14]. In a larger study by the same group, EBV encephalitis occurred in 12% of patients with DNAemia exceeding 1000 copies/mL [15]. Wu et al. reported a three‐year cumulative incidence of EBV encephalitis of 5.6% ± 1.4% among allogeneic HCT recipients [16].

In contrast, other studies have described lower incidences of EBV neurological disease. Schmidt‐Hieber et al., in an analysis of more than 2500 allogeneic HCT recipients, documented 32 cases of viral encephalitis for an overall incidence of 1.2% (95% CI, 0.8–1.6%). In that series, HHV‐6 (9 cases) and EBV (6 cases) were the most frequently identified pathogens [17]. Another study of 263 allogeneic HCT recipients found a three‐year cumulative incidence of EBV encephalitis/myelitis of 1.6% ± 0.8% [18]. The frequency observed in our study (2.2%) is therefore consistent with the range reported in the literature [14, 15, 16, 17, 18].

Several factors may account for the wide variation in published incidence rates of EBV encephalitis, including heterogeneity of patient populations with respect to EBV risk, monitoring schedules, the commercial qPCR platforms employed, absence of standardized EBV DNA cutoffs for preemptive intervention, diagnostic promptness, and timeliness of rituximab administration, among others.

Response rates to preemptive rituximab are reported at approximately 60%–70%, increasing to 84% when combined with IS reduction [10, 11]. Similarly, improved survival and lower mortality rates have been documented when IS tapering is added to rituximab [11, 19], underscoring the critical role of IS reduction in the management of EBV complications. According to some studies, IS reduction alone as preemptive therapy achieved a success rate of about 68% [20, 21].

In the present study, we aimed to demonstrate that IS reduction alone could control the majority of EBV reactivation episodes, with rituximab reserved for cases unresponsive to IS tapering, without compromising OS or NRM. Consistent with this approach, rituximab was required in only 12 of 178 patients (6.7%) with EBV reactivation, the frequencies of PTLD or EBV end‐organ disease were comparable to published data, and a 75% response rate among patients treated for EBV encephalitis.

The restrictive rituximab policy applied at our center did not significantly affect OS, NRM, or relapse rates between patients with and without EBV reactivation. Importantly, our active surveillance program—including close monitoring of EBV viral load kinetics and prompt laboratory alerts—was essential to enable rapid clinical intervention.

Our study has several limitations. First, it was a retrospective, uncontrolled, single‐center analysis, which may limit the generalizability of the findings. Second, donor/recipient EBV serological status and rituximab‐related toxicities were not systematically assessed. Third, our strategy required close coordination between the laboratory and the transplant infectious diseases team, with real‐time alerts for every ≥1‐log increase in EBV qPCR. Fourth, a potential drawback of IS reduction as a means to control EBV is the onset or worsening of GVHD.

Although our IS reduction strategy proved as effective and safe as preemptive rituximab, EBV reactivation emerged as a risk factor for chronic GVHD. This association may reflect the consequences of accelerated IS tapering rather than EBV itself. Unfortunately, the study design did not allow us to determine whether the increased risk was attributable to EBV reactivation per se, the rapid IS reduction in response to rising viral load, or donor EBV serostatus—which was not available in our series. Notably, donor EBV seropositivity has also been associated with higher risks of both acute and chronic GVHD in previous studies [22, 23].

Finally, we would like to emphasize that rituximab increases the risk of infections and interferes with vaccine responses for a prolonged period. Several studies have documented associations between rituximab use and secondary hypogammaglobulinemia, impaired humoral immunity, and a higher incidence of infectious complications, as well as diminished vaccine efficacy [24, 25, 26]. In a recent evaluation of the benefits and risks of rituximab—whether given prophylactically, preemptively, or therapeutically, the authors reported associations with hypogammaglobulinemia, neutropenia, lymphopenia, increased infection burden, a higher incidence of fatal infections, increased NRM, and higher overall mortality, and recommended that centers adopting preemptive rituximab consider applying a higher EBV DNAemia threshold to minimize unnecessary exposure [4].

Rituximab is an important treatment option across many specialties. However, the strength of evidence for preemptive use after EBV reactivation in allogeneic HCT remains moderate and is largely based on uncontrolled studies [8]. Therefore, from the transplant infectious diseases specialist's perspective, the benefits and risks of this recommendation should be carefully balanced, and consideration should be given to adopting a higher EBV DNAemia threshold to avoid unnecessary rituximab administration, as suggested by other authors [4].

## Author Contributions

ABCS extracted the data, analyzed the results, prepare the tables and figures, and wrote the manuscript. AJS extracted the data, conducted the statistical analysis, prepare the figures, provided the feedback on the report. ACFS extracted the data, provided the feedback on the report. JRPM, LPZ, LCSO performed the EBV qPCR and extracted the laboratory data. IC, FRB, ERPD, JSS, MPS assisted the patients, provided the feedback on the report. VARC analyzed the results, provided the feedback on the report. cmm designed the study, conducted the research, analyzed the results, prepare the tables and figures, and wrote the manuscript.

## Funding

The study was partially supported by Fundação de Amparo à Pesquisa do Estado de São Paulo (FAPESP)—grant 2009/16364‐6.

## Conflicts of Interest

the authors declare no conflicts of interest.
